# Tailored progesterone for luteal phase support: the way to go

**DOI:** 10.5935/1518-0557.20230031

**Published:** 2023

**Authors:** Garima Patel, Richa Vatsa, Aryan Kashyap

**Affiliations:** 1 Department of Obstetrics and Gynecology, All India Institute of Medical Sciences, New Delhi, India

We read the recently published article by Scheffer *et al.* (2023) with great interest. Their study is one of the few to evaluate the impact of serum progesterone level in luteal phase on pregnancy rates in fresh blastocyst transfer in antagonist cycles. The study also provides an insight into an optimal progesterone level on the day of blastocyst transfer with day-5 of progesterone supplementation (P4d5+) and gives a cut-off of ≥10 ng/ml to be associated with a significantly higher pregnancy rate. This could help us provide a more tailored progesterone supplementation for our patients, as it is well known that progesterone should be used with great caution and only when indicated (O’Brien & Lewis, 2016).

As much informative and thought-provoking as this article has been, we as readers would like to enquire the authors regarding some of our queries, that would help us in better understanding of the study:

- There is mounting evidence regarding the “ceiling effect” of progesterone wherein high levels of progesterone on the day of embryo transfer have shown to decrease live birth rates associated with blastocyst transfer in a Frozen embryo transfer. Recent studies (Alyasin *et al*., 2021) have shown that progesterone levels more than 32.5 ng/dL are associated with lower live birth rates. It would be of great interest for us readers to know, if detrimental effects of high serum progesterone levels were documented in their study.
- The authors of the study have taken a cut-off of 10ng/ml, based on prior studies (Cédrin-Durnerin *et al*., 2019) to divide their patients into groups A and B, after which a correlation analysis was performed. However, in their study, Cédrin-Durnerin *et al*. (2019) performed a day 2/3 or a blastocyst transfer in a frozen cycle contrary to the current study, where blast transfer was done in a fresh cycle. Thus, should cut-off from such a heterogenous study be validated for the current study, is a matter of concern for us readers.
- Most studies in recent times (Cédrin-Durnerin *et al*., 2019; Alyasin *et al*., 2021) have used a cumulative dose of 600 mg micronized progesterone given intravaginal while establishing the cut-off values for optimum serum progesterone. The protocol for the current study used a cumulative dose of 1200 micronized progesterone given intravaginal. Since we strive to tailor the dose of progesterone to an optimum low due to its adverse effects and a possibility of adverse pregnancy rates in patients with high serum progesterone levels, a lower dose of vaginal progesterone would have been more suitable.
- The predictive value of serum progesterone on the day of blastocyst transfer for pregnancy rates should have been calculated by a Receiver operating characteristic (ROC) curve analysis. This would have further consolidated the evidence regarding an optimum progesterone cut-off value.
- A glance at the data presented in table 2, such as the AMH values suggest the data to be skewed. We suggest that it would be more appropriate if the normality of data was checked, and data be presented in the form of medians and inter-quartile ranges.
- Recent guidelines by the CDC (2019) states that ICSI is associated with an increased risk of chromosomal abnormalities, autism, intellectual disabilities, and birth defects, compared to conventional IVF. Therefore, unindicated ICSI is not advisable.
- Since progesterone is started on the day of pickup, in most of the patients, we expect the endometrium to become luteinised. Therefore, the endometrium on the day of embryo transfer might have been trilaminar on the day of trigger, but we do expect the endometrium to become luteinised and diffuse on the day of embryo transfer in almost all patients.
- One of the important factors that has been shown to increase perinatal and maternal morbidity in ART is multifetal gestation. The Practice Committee of the American Society for Reproductive Medicine and the Practice Committee for the Society for Assisted Reproductive Technologies (2021) guidelines recommend that a single transfer of euploid / favourable blastocyst is preferred. Thus, we as readers would like to enquire if the practice of two blastocyst transfers is a routine at the author’s institute, as we now universally follow a single embryo transfer approach.
- High values of progesterone on the day of trigger have also shown to have detrimental effects on the outcomes of fresh IVF cycles (Irani *et al*., 2020). In the current study, even though an attempt to measure progesterone on the day of trigger (P4dhCG) was made, it was not studied as an independent factor affecting pregnancy outcomes in blastocyst transfer. It would be quite enlightening for the readers if the relationship between P4dhCG (as an independent variable) and embryo transfer outcomes could also be elucidated.

We appreciate the authors for their effort at defining an optimum progesterone level predictive of successful IVF outcome in blastocyst transfer. However, we as infertility specialists must use progesterone at tailored optimum doses to prevent adverse outcomes associated with progesterone use. Adequately powered, large prospective studies will be helpful in the near future on the current topic.
